# Are Canadian General Internal Medicine training program graduates well prepared for their future careers?

**DOI:** 10.1186/1472-6920-6-56

**Published:** 2006-11-17

**Authors:** Sharon E Card, Linda Snell, Brian O'Brien

**Affiliations:** 1 Department of Medicine, University of Saskatchewan, Saskatoon, Canada; 2 Department of Medicine, McGill University, Montreal, Canada; 3 Faculty of Medicine and Dentistry, University of Alberta, Edmonton, Canada

## Abstract

**Background:**

At a time of increased need and demand for general internists in Canada, the attractiveness of generalist careers (including general internal medicine, GIM) has been falling as evidenced by the low number of residents choosing this specialty. One hypothesis for the lack of interest in a generalist career is lack of comfort with the skills needed to practice after training, and the mismatch between the tertiary care, inpatient training environment and "real life". This project was designed to determine perceived effectiveness of training for 10 years of graduates of Canadian GIM programs to assist in the development of curriculum and objectives for general internists that will meet the needs of graduates and ultimately society.

**Methods:**

Mailed survey designed to explore perceived importance of training for and preparation for various aspects of Canadian GIM practice. After extensive piloting of the survey, including a pilot survey of two universities to improve the questionnaire, all graduates of the 16 universities over the previous ten years were surveyed.

**Results:**

Gaps (difference between importance and preparation) were demonstrated in many of the CanMEDS 2000/2005^® ^competencies. Medical problems of pregnancy, perioperative care, pain management, chronic care, ambulatory care and community GIM rotations were the medical expert areas with the largest gaps. Exposure to procedural skills was perceived to be lacking. Some procedural skills valued as important for current GIM trainees and performed frequently (example ambulatory ECG interpretation) had low preparation ratings by trainees. Other areas of perceived discrepancy between training and practice included: manager role (set up of an office), health advocate (counseling for prevention, for example smoking cessation), and professional (end of life issues, ethics).

**Conclusion:**

Graduates of Canadian GIM training programs over the last ten years have identified perceived gaps between training and important areas for practice. They have identified competencies that should be emphasized in Canadian GIM programs. Ongoing review of graduate's perceptions of training programs as it applies to their current practice is important to ensure ongoing appropriateness of training programs. This information will be used to strengthen GIM training programs in Canada.

## Background

In Canada Internal Medicine training is four years in length. The first three years of training are considered "core" after which the resident enters either a subspecialty or a general internal medicine (GIM) training program. Canadian GIM (post core) training programs are a variety of lengths (one to two years) and formats. The Royal College of Physicians and Surgeons of Canada have not yet recognized GIM as a distinct entity in Canada, with the fourth year of training remaining officially under the auspices of internal medicine (IM). In many programs, training in the GIM 4^th ^and 5^th ^years has been a potpourri of training, not necessarily reflecting the needs of the community, or the experience of previous trainees. As GIM training has not traditionally been thought of as a distinct entity the development of a consistent national curriculum, objectives and educational programs for those who will practice GIM has not occurred. We hypothesized that this may leave residents feeling unprepared for their eventual careers. A review of curriculum and objectives specifically aimed at training general internists was felt indicated.

Since a survey in 1989 [1] there has been little information added to the knowledge base of the practice pattern and scope of practice of the General Internist trained and practicing in Canada. We evaluated whether graduates of current training programs felt their training programs prepared them adequately for practice. This information will then be used to develop national objectives and curriculum for the training of General Internists in Canada.

## Methods

### Study population

The GIM program directors (those responsible for the PGY 4 +/- 5 residents specifically in GIM training programs) were asked to forward the names of trainees who had participated in their training programs over the years 1993 to 2001. In all but one university this was an individual separate from the program director responsible for the "core, PGY 1 to 3" trainees. All universities forwarded the names of their graduates. In some cases the training program was able to supply addresses of the individuals but in many cases they could not. The investigators mailed the survey to the names provided by the program directors after attempting to find addresses. The complete population was studied.

### Survey design

The survey was developed after review of the literature. Modifications were made after feedback was obtained from the Canadian Society of Internal Medicine (CSIM) Council, CSIM Education Committee, Canadian Association of Internal Medicine Program Directors (the program directors responsible for the first three years of training and the 4^th ^overlap year in any subspecialty and GIM) and the General Internal Medicine Program Directors of Canada (those responsible specifically for the 4^th ^and 5^th ^year training programs in GIM). The survey was piloted at two universities – Dalhousie University in Halifax, Nova Scotia and the University of Saskatchewan, Saskatoon, Saskatchewan. Subsequently further changes to the survey were made based on the results. A copy of the final survey is available on the web site (see Additional File 1). This was a mailed survey with two reminders.

### Data collection

Graduates were asked about their **preparation rating **for various skills and elements of practice (How well prepared for practice did you feel in each of these areas at the end of your GIM (post core) training? 1 = not at all prepared to 5 = well prepared) and **importance rating **for various skills and elements of practice (How important is each of these for GIM training now? 1 = not at all and 5 = very). They were also asked about procedures they performed and how frequently. Data were entered into Microsoft ACCESS^® ^and EXCEL^® ^spreadsheets.

### Data analysis

Data is presented as percentage of respondents answering 1 and 2 or 4 and 5 on the Likert scale for preparation and importance ratings. Data is presented as percentage of respondents performing the procedure at least once monthly.

### Ethics

The research was reviewed and approved on ethical grounds by the University of Saskatchewan Advisory Committee on Ethics in Behavioral Science Research (January 2002).

## Results

Of 542 surveys sent, 71 were returned with a wrong address. 183 were completed for a response rate of 39 %.

Table 1 illustrates the characteristics of practice of the respondents who were asked these questions. Due to changes in the survey after the pilot survey some respondents were asked different demographic questions – the responses in table one are reported as percentage for all of those who were asked the question. The year of graduation for all of the respondents was fairly evenly split from 1.14 % graduating in 1991 to 20.00 % graduating in 2001. The majority (greater than 64 %) graduated in 1998 or later. 73.22 % had graduated from a one year general internal medicine program and 9.29 % from a two year program. Thirty-five percent had subspecialty training following their general internal medicine training; nine percent were still in further sub-specialty training at the time of the survey.

When asked the question "How important is knowledge in each of these medical disciplines for GIM training now?" the following percentage of respondents answered either 4 or 5 (with 5 = very important) on the Likert scale: Cardiology (99 %); Respirology (92 %); Infectious Diseases (87 %); Nephrology (86 %); Critical Care (86 %) and Endocrinology (83 %). Those indicating 4 or 5 (with 5 = well prepared) on the preparation rating ("How well prepared for practice did you feel in each of these medical disciplines?") had a similar rank order: Cardiology (92 %); Respirology (85 %); Nephrology (79 %); Infectious Diseases (76 %); and Critical Care (74 %). The largest gaps between the percentage of respondents indicating 4 or 5 for importance and preparation were for: Clinical Pharmacology (31.33 %; 67 % answering 4 or 5 for importance;36 % for preparation); Neurology (26.54 %; 76 % answering 4 or 5 for importance; 49 % for preparation); Endocrinology (25.64 %; 83 % answering 4 or 5 for importance; 57 % for preparation) and Palliative Medicine (25.04 %; 52 % answering 4 or 5 for importance; 27 % for preparation).

Table 2 illustrates the differences between importance and preparation for various skills expressed as percentage of respondents indicating 1 or 2 on the Likert scale versus 4 or 5. Many areas within the CanMEDs competencies have a large difference between importance and preparation particularly medical problems of pregnancy, perioperative care, counseling for prevention (example smoking) and set up of an office. The areas with a gap of greater than twenty percent for preparation versus importance rating are highlighted in bold on Table 2.

**Figure 1:**
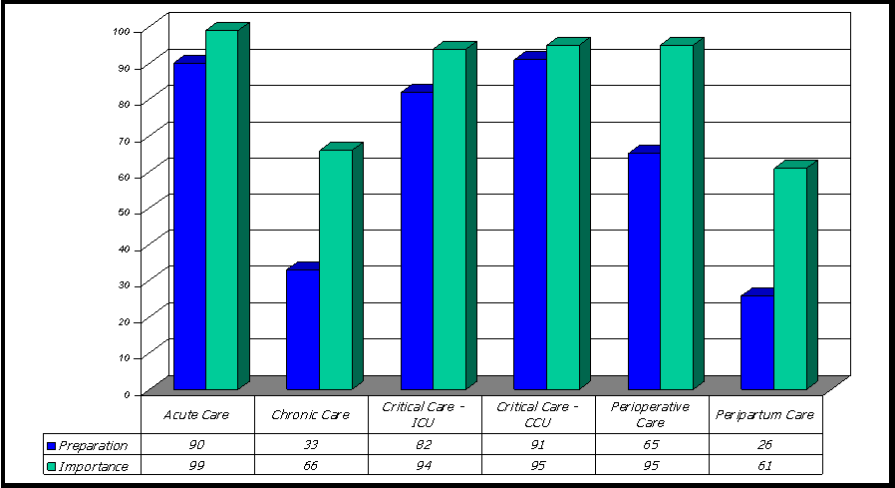
Needs met and importance of various content areas of training programs. Expressed as percentage of respondents answering 4 or 5 on the Likert Scale.

Figure 1 (see Figure 1) illustrates preparation and importance for specific content areas in GIM training programs. Again reinforced is the discrepancy between preparation and importance for peripartum care, perioperative care, as well as for chronic care. Acute care and critical care are both felt to be very important by graduates and they did feel prepared in these areas. Exposure to procedural skills was an area of discrepancy with 90 % of respondents indicating it as important (4 or 5 on the Likert scale) but only 58 % felt prepared (4 or 5 on the Likert scale). Several areas of the administrative structure of training programs (clear goals and objectives, flexibility, responsibility separate from that of a third year resident, timely on-going evaluation) all were felt to need improvement (discrepancy between preparation and importance, data not shown).

Only 47.43 % of respondents felt that their programs met their needs in terms of community GIM rotations (47.43 % responding 4 or 5 on the Likert scale) which was much lower than the indicated importance (83.64 % responding 4 or 5 on the Likert scale). For ambulatory care rotations there was also a large discrepancy (53.93 % for needs met versus 91.67 % for importance). Inpatient rotations were rated by 84.57 % of respondents as 4 or 5 on the preparation rating and by 75.30 % as 4 or 5 on the importance rating.

Table 3 illustrates the difference between importance and preparation for procedural skills with each being represented as percentage of respondents answering 4 and 5 or 1 and 2 on the Likert Scale. Only five skills (ACLS/CPR; lumbar puncture, ambulatory ECG interpretation; endotracheal intubation; paracentesis) were identified by greater than 80 % of the respondents as being quite important (rated as 4 or 5 on the Likert scale). For those skills for which the majority (greater than 60 % of respondents) felt were important (rated as 4 or 5 on the Likert scale) there was a greater than 15 % discrepancy between those answering 4 or 5 on the Likert scale for preparation and importance for the following skills: ambulatory ECG interpretation; endotracheal intubation; exercise stress testing; temporary pacemaker.

Table 4 shows the number of respondents performing each procedural skill at least once per month, while table 5 shows the number of times a procedure is done for those that are considered most important. Fourteen procedural skills were reported by greater than 85 % of respondents as being performed zero times per month; these include: allergy testing, bronchoscopy, colonoscopy, echocardiography, esophagogastroscopy, hemodialysis, indirect laryngoscopy, liver biopsy, peritoneal dialysis, pleural biopsy, renal biopsy, sigmoidoscopy, sputum gram stain, thyroid fine needle biopsy.

## Discussion

This study was designed to try to look for the first time in ten years at any gaps in the training of general internists in Canada. Information from the United States indicates that there is a discrepancy between the practice patterns of practising internists and what they perceived their training programs prepared them for [2-6]. There are major differences in the practice of General Internal Medicine between the United States and Canada [7-9]. These include both the nature of practice (primary care in the US versus referred specialty care in Canada) and the length and kind of training. Training in the United States is generally a year shorter and based more heavily in the ambulatory setting. It is therefore important to have Canadian data on which to base the content and structure of Canadian programs.

This study like those in the United States [2,6,10-16] demonstrates that graduates of Canadian GIM programs note gaps between preparation and importance for several procedural skills. This is particularly true for several ambulatory based procedures (example ambulatory ECG monitoring). Consideration should be given to having further emphasis on such procedures in GIM training programs. Canadian general internists are perceived to perform many procedural skills in practice. Two studies have looked at the procedural skills performed by Canadian General Internists [17,18], without solid conclusions as to which procedural skills would be particularly needed by graduates of a General Internal Medicine training program.

Many procedures are performed by very few individuals. We would therefore propose that flexible, individually tailored programs would best fit the needs of individual residents and presumably society. It would be reasonable to expect that all trainees in GIM training programs learn those skills that at least 50 % of respondents utilize at least once per month (ambulatory ECG interpretation, paracentesis, lumbar puncture, ACLS/CPR, cardioversion, mechanical ventilation, articular drainage, endotracheal intubation, hemodynamic monitoring, exercise stress testing, transthoracic pacing). This would be in addition to the eight skills in the Royal College of Physicians and Surgeons of Canada Internal Medicine objectives (central venous catheter insertion; lumbar puncture; peripheral arterial catheter insertion; abdominal paracentesis; endotracheal intubation; thoracentesis; knee joint aspiration; and electrocardiographic interpretation). For other skills an individualized training program for each resident taking into account their eventual needs and where they are likely to practice is proposed versus a long list of required competencies for each resident.

Tailoring of training to the various contexts (example in hospital, academic, outpatients) within which general internists may practice was suggested by the SGIM task force in the United States [19,20] along with the suggestion that general internal medicine residents should have options to tailor their final 1 to 2 years to fit their practice goals, earning a certificate of added qualification in generalist fields [19,20]. Although much less has been written about the fit of general internal medicine into the Canadian health care system [7-9] we would propose a similar training pattern for Canadian general internists.

In 1989 Linda Snell undertook a survey of practicing Internists in Canada [1]. There were perceived deficiencies in training in ambulatory care, in the management of complex disorders over time, in management of geriatric patients and those with psychosocial problems. Other areas of perceived deficiency included procedures, especially ICU and endoscopy, teaching skills, continuing self-education skills as well as administration and office management. There was over preparation in other areas. Shamekh and Snell in a survey of graduates of one University found that the current ambulatory care structure in their training program did not satisfy the needs of the graduates [21].

Like these previous studies our data illustrates a gap between importance and preparation for training particularly in the areas of ambulatory care and chronic disease management. There is also a gap perceived for preparation for perioperative care and medical disorders of pregnancy. This is particularly disturbing in that in Canada these have been 'traditionally' felt to be key aspects of GIM practice. Ambulatory care and community general internal medicine are the rotations being pointed out as needing to be strengthened.

In other specialties evidence has shown a gap in training in such non-clinical skills as health service delivery and non-clinical roles [22]. Whether this is true in Canadian GIM programs was suspected by the authors but not documented in the literature. In New Zealand one of these gaps was overcome by developing a national forum for all registrars in several non-clinical skills [22]. Our study shows a dramatic discrepancy between preparation for set-up of an office and importance suggesting that areas of instruction outside the CanMEDs role of medical expert need to be strengthened, perhaps with such a national forum. A recent study looking at paediatric residency programmes in Canada [23] also indicated less than adequate preparation for manager of an office practice.

There are limitations to this study. The response rate is low and was hampered by trying to find individuals in programs that are not registered by the Royal College of Physicians and Surgeons of Canada directly. This made it difficult to find many of the addresses and respondents. As well as these individuals may have been registered in a 4^th ^year program with the intent to eventually go into a subspecialty (approximately 35 %) this information is contaminated by some individuals who went on to become subspecialists. To avoid this we could have surveyed those individuals who are currently practicing GIM in Canada and sought out these individuals either through communities directly or through the Canadian Society of Internal Medicine to which many general internists in Canada belong. Many of the general internists particularly in rural community areas in Canada are not trained in Canada and many come from other countries. As the intent of this study was to look specifically at the discrepancy between preparation and importance for those who have trained in Canadian training programs we chose to identify our respondents this way.

As with many survey studies our results are individual's perceptions only. Individuals may not feel prepared in a topic but may actually able to practice the competency quite well. This data is unknown and is not captured in this study. We do want to take into account the perspectives of those practicing GIM as we develop the objectives for GIM in Canada thus although it is not a gold standard for competence we do believe the perspectives of these respondents is important. As the number of respondents in each year cohort is small we are unable to assess whether more recent graduates feel more confident in areas such as office management which may be emphasized to a greater extent since the introduction of the Royal College of Physicians and Surgeons CanMeds competency framework in 2000.

## Conclusion

This information will be one of the sources used by the Working Group in General Internal Medicine (Royal College of Physicians and Surgeons of Canada) to create national objectives and standards for GIM. We propose that:

1. There are core competencies that each general internist should learn, and there should be national standards for these. These should include:

a. Continued excellence in training in acute care.

b. Enhanced training in medical problems of pregnancy.

c. Strengthened training in ambulatory and community GIM.

d. Improved training in manager and health advocate CanMEDs roles.

2. Beyond these core competencies, training needs to be flexible (including length of training) and adapted to each trainee for their anticipated role in health care delivery. Optional competency based modules should be developed (example specialty interest, medical education).

3. Access to training in procedural skills needs to be ensured, improved, individualized and a rigorous evaluation process developed. Advanced procedural skills training would only be undertaken by those who need these skills to meet societal needs.

## Competing interests

All authors are members of the Canadian Society of Internal Medicine which provided support for this study.

## Authors' contributions

SEC, LS and BO'B were involved in the development and piloting of the survey instrument, design of the study, interpretation of the data and writing of the manuscript. All authors read and approved the final manuscript.

## Pre-publication history

The pre-publication history for this paper can be accessed here:



## Supplementary Material

Additional File 1 Survey – Canadian General Internal Medicine Training. Copy of survey instrument used. Click here for file

## Figures and Tables

**Table 1:** Demographics of respondents for whom data is available.

	**Number**	**Percentage**
**PRACTICE TYPE:**		
Community-based without a university appointment	53	37.32 %
Community-based with a university appointment	27	19.01 %
University-based full time	38	26.76 %
Hospital-based	75	52.82 %
Office-based	11	7.75 %
Both Hospital and Office – based.	44	30.99 %
**CATCHMENT AREA:**		
Rural	17	11.97 %
Small (< 100,000) urban	21	14.79 %
Large (> 100,000) urban	92	64.79 %
> 100 km from tertiary care centre	14	9.86 %
**CLINICAL PRACTICE:**		
> 75 % General Internal Medicine	101	71.13 %
**SPECIAL CLINICAL INTEREST IN:**		
ICU	38	26.76 %
CCU	31	21.83 %
Cardiology	52	36.62 %
Diabetes	30	21.13 %
Gastroenterology	10	7.04 %
Endocrinology	15	10.56 %
Respirology	15	10.56 %
Medical problems in pregnancy	17	11.97 %
Pre-operative consultation	41	28.87 %
**OTHER TRAINING OR INTEREST:**		
Clinical epidemiology	25	17.61 %
Medical education	35	24.65 %
Public health	3	2.11 %
Health services	4	2.82 %
Ethics	6	4.23 %
Health Administration	8	5.63 %

**Table 2:** Preparation and importance in various skills.

	Preparation % 1 or 2	Preparation % 4 or 5	Importance % 1 or 2	Importance % 4 or 5
***MEDICAL EXPERT***				
History taking	0.00 %	100.00 %	0.00 %	99.40 %
Physical examination	0.00 %	98.31 %	0.00 %	99.39 %
Diagnosis of undifferentiated illness	1.14 %	91.48 %	0.00 %	98.17 %
Management of chronic illness	3.98 %	80.11 %	1.22 %	95.12 %
Care of multi-system disease	0.56 %	94.35 %	0.00 %	99.40 %
Care of critically ill patients	2.29 %	84.57 %	0.00 %	96.95 %
Develop problem-solving	0.56 %	94.92 %	0.00 %	99.40 %
Medical problems of pregnancy	21.59 %	**37.50 %**	3.64 %	**75.76 %**
Peri-operative care	9.04 %	**66.10 %**	0.00 %	**92.77 %**
Therapeutics	1.14 %	80.57 %	0.61 %	95.73 %
Pain management	33.33 %	**27.12 %**	7.32 %	**74.39 %**
Recognize treatable rare diseases	9.04 %	55.93 %	4.22 %	74.70 %

***COMMUNICATOR***				

Communication skills	2.82 %	87.01 %	0.60 %	96.99 %
Consultation skills	2.26 %	81.92 %	0.00 %	98.19 %

***COLLABORATOR***				

Collaborate with non-physician caregivers	14.04 %	64.04 %	2.41 %	83.13 %

**MANAGER**				

Administration skills	57.06 %	**16.38 %**	24.85 %	**42.42 %**
Set-up of an office	84.83 %	**2.81 %**	15.76 %	**69.09 %**

***HEALTH ADVOCATE***				

Choose cost effective treatments	20.79 %	**45.51 %**	4.22 %	**78.92 %**
Counsel regarding smoking	52.54 %	**23.73 %**	8.48 %	**65.45 %**
Counsel regarding exercise	45.20 %	**25.42 %**	7.27 %	**70.91 %**
Counsel regarding HIV testing	32.20 %	**38.98 %**	12.12 %	**60.61 %**
Counsel about domestic violence	74.58 %	**9.04 %**	29.70 %	**36.36 %**
Counsel about substance abuse	58.19 %	**16.95 %**	21.34 %	**50.00 %**

***SCHOLAR***				

Research skills	40.68 %	24.29 %	23.49 %	36.75 %
Develop life-long learning skills	2.27 %	85.23 %	0.00 %	98.18 %
Critical appraisal	7.34 %	**66.10 %**	1.81 %	**87.35 %**
Participate in quality assurance	43.82 %	**22.47 %**	12.65 %	**58.43 %**
Teaching skills	5.08 %	67.80 %	1.81 %	80.12 %

***PROFESSIONAL***				

End-of-life issues	19.10 %	**56.18 %**	3.61 %	**83.73 %**
Ethics	14.12 %	**55.37 %**	3.61 %	**78.31 %**
Compliance issues	26.55 %	**36.72 %**	3.03 %	**81.21 %**

Expressed as % of respondents indicating 1 or 2 on the Likert Scale versus 4 or 5. Those with a gap of > 20 % between preparation and importance are high lighted in bold.

**Table 3:** Preparation and importance for procedural skills.

	**Preparation % 1 or 2**	**Preparation % 4 or 5**	**Importance % 1 or 2**	**Importance % 4 or 5**
**ACLS/CPR**	0.56 %	95.0%	0.60 %	92.22 %
**Allergy testing**	88.20 %	3.37 %	88.03 %	1.82 %
**Ambulatory ECG interpretation**.	11.80 %	73.03 %	4.22 %	89.76 %
**Articular Drainage**	25.14 %	44.13 %	11.90 %	54.76 %
**Bone marrow interpretation**	60.57 %	16.00 %	57.67 %	19.02 %
**Bronchoscopy**	86.29 %	6.86 %	63.80 %	12.88 %
**Capillary Blood Glucose**	42.11 %	42.11 %	48.48 %	39.39 %
**Cardioversion**	17.32 %	58.66 %	5.42 %	69.88 %
**Chest tube insertion**	47.46 %	34.36 %	18.07 %	51.20 %
**Colonoscopy**	86.93 %	6.82 %	67.07 %	12.20 %
**Echocardiography**	91.48 %	3.98 %	61.21 %	20.61 %
**Endotracheal intubation**	13.56 %	62.71 %	3.59 %	83.23 %
**Esophagogastroscopy**	84.00 %	8.57 %	61.96 %	16.56 %
**Exercise stress testing**	27.12 %	50.85 %	8.48 %	73.33 %
**Foley catheter insertion**	44.74 %	34.21 %	33.33 %	33.33 %
**Hemodialysis**	61.93 %	13.07 %	59.04	13.25 %
**Hemodynamic monitoring**	10.80 %	64.20 %	6.63 %	68.07 %
**Indirect laryngoscopy**	90.40 %	2.82 %	73.94 %	4.85 %
**Liver biopsy**	86.44 %	5.08 %	79.52 %	7.83 %
**Lumbar puncture**	2.22 %	85.56 %	1.79 %	91.07 %
**Mechanical ventilation**	7.82 %	63.69 %	1.82 %	76.97 %
**Microscopic exam of urine**	36.46 %	36.46 %	25.44 %	43.79 %
**Paracentesis**	2.78 %	87.22 %	1.80 %	81.44 %
**Peripheral smears – Interpretation**	53.63 %	24.02 %	43.03 %	28.48 %
**Peritoneal dialysis**	67.22 %	6.67 %	63.47 %	7.19 %
**Pleural biopsy**	81.67 %	5.56 %	73.49 %	7.83 %
**Renal biopsy**	93.89 %	1.67 %	90.96 %	2.41 %
**Sigmoidoscopy**	80.00 %	12.22 %	47.27 %	27.27 %
**Sputum Gram Stain**	69.06 %	12.71 %	56.80 %	20.12 %
**Steroid Injections (Non-articular)**	56.50 %	23.73 %	43.64 %	27.88 %
**Temporary Pacemaker Insertion**	37.99 %	39.66 %	19.16 %	61.08 %
**Thyroid Fine Needle Biopsy**	79.89 %	7.82 %	50.30 %	17.96 %
**TPN Initiation**	49.16 %	21.79 %	23.95 %	38.32 %
**Tracheostomy Tube Change**	71.67 %	15.00 %	51.48 %	20.71 %
**Transthoracic Pacing**	19.44 %	54.44 %	9.47 %	65.68 %

Preparation and Importance expressed as percentage of respondents answering 1 and 2 or 4 and 5 on the Likert Scale.

**Table 4:** Number of respondents performing procedural skills at least once per month.

	**Percentage of Respondents** **Performing Procedure** **At Least Once Per Month**
**Ambulatory ECG Interpretation**	88 %
**Paracentesis**	84 %
**Lumbar Puncture**	81 %
**ACLS/CPR**	79 %
**Cardioversion**	70 %
**Mechanical Ventilation**	68 %
**Articular Drainage**	65 %
**Endotracheal Intubation**	64 %
**Hemodynamic Monitoring**	63 %
**Exercise Stress Testing**	55 %
**Transthoracic Pacing**	54 %
**TPN Initiation**	49 %
**Temporary Pacemaker Insertion**	47 %
**Microscopic Examination of Urine**	39 %
**Chest Tube Insertion**	38 %
**Steroid Injections (non-articular)**	36 %
**Peripheral Smears Interpretation**	32 %
**Capillary Blood Glucose**	27 %
**Bone Marrow Interpretation**	25 %
**Tracheostomy Tube Change**	23 %
**Foley Catheter Insertion**	22 %
**Bronchoscopy**	14 %
**Esophagogastroscopy**	13 %
**Thyroid Fine Needle Biopsy**	13 %
**Hemodialysis**	12 %
**Sigmoidoscopy**	11 %
**Sputum gram stain**	11 %
**Echocardiography**	10 %
**Peritoneal dialysis**	10 %
**Colonoscopy**	9 %
**Indirect laryngoscopy**	6 %
**Liver biopsy**	5 %
**Allergy Testing**	4 %
**Pleural Biopsy**	3 %
**Renal Biopsy**	1 %

**Table 5:** For the 7 skills that greater than 70 % of respondents felt were Important/Very Important (4 or 5 on the Likert Scale) to learn – How many are done in practice?

	**0**	**<5**	**5–9**	**10–14**	**15+**
**ACLS/CPR**	20.79 %	59.55 %	14.04 %	3.37 %	2.25 %
**Lumbar Puncture**	19.34 %	73.48 %	5.52 %	0.55 %	1.10 %
**Ambulatory ECG Interpretation**	12.36 %	8.99 %	9.55 %	7.87 %	61.24 %
**Endotracheal Intubation**	36.52 %	50.00 %	10.11 %	2.25 %	1.12 %
**Paracentesis**	15.56 %	70.56 %	11.67 %	1.11 %	1.11 %
**Mechanical Ventilation**	32.40 %	35.75 %	16.76 %	4.47 %	10.61 %
**Exercise Stress Testing**	44.63 %	8.47 %	6.21 %	4.52 %	36.16 %
