# Supplementary material for: Are Canadian General Internal Medicine training program graduates well prepared for their future careers?
Source: BMC Med Educ. 2006 Nov 17;6:56. doi: 10.1186/1472-6920-6-56 (PMC1664564; doi:10.1186/1472-6920-6-56)
Supplement: Additional File 1 — Survey – Canadian General Internal Medicine Training. Copy of survey instrument used. [file 1472-6920-6-56-S1.doc]

**To: Recent graduates of Canadian General Internal Medicine programmes**

You have been identified through your programme director at the university where your completed your post-graduate training in Internal Medicine.

Many thanks for taking the time to fill out this survey! We are very interested in the perspectives of those who have trained in Canadian General Internal Medicine Training Programs and in how this training has prepared you for your current practice. We are going to use it for development of a modernized curriculum for the General Internal Medicine/Post-core (Fourth Year) training programme. It should take ~ 20 – 30 minutes to fill out.

Sincerely

Sharon Card, Brian O’Brien and Linda Snell

In recognition of your valuable time, we invite you to enter a draw for your choice of:

registration fee for the CSIM annual meeting in Toronto, May 7-10, 2003, or

annual membership fee in the CSIM; or

a Personal Digital Assistant

To enter the draw, please complete the information below. If you are interested in receiving a copy of the final report for this study, please indicate this as well.

To ensure your anonymity, this form will be detached from your questionnaire before your responses are analyzed.

Name

Address

Telephone: Fax: email:

Prize choice (circle): meeting registration CSIM membership PDA

Canadian Society of Internal Medicine current member (circle): yes no

Please send me a copy of the final report of this study yes no

All questionnaire responses will remain confidential and only group data will be reported. Upon receipt of your completed questionnaire and identification form, secretarial staff will immediately separate them, and will use the form to record that you have responded. The study team will not have access to this information.

**1. Preparation for Practice.** There are two components to each of the next five questions:

**Preparation score:** how **prepared** did you feel in each area at the end of your GIM (post Core) training?

scale: 1 = not at all prepared 5 =very well prepared

**Importance score:** how **important** do you currently feel each area is for those training in GIM (post Core) at the present time?

scale: 1 **=** not at all important 5 = very important

1. **Medical Disciplines**

## Check score in each column

|  | 1. How well **prepared** for practice did you feel in each of these medical disciplines? | 2. How **important** is knowledge in each of these medical disciplines for GIM training now? |
| --- | --- | --- |
|  | **1 = not at all 5 = very well** | **1 = not at all 5 = very** |
|  | **1 2 3 4 5** | **1 2 3 4 5** |
| Cardiology | □ □ □ □ □ | □ □ □ □ □ |
| Clinical Pharmacology | □ □ □ □ □ | □ □ □ □ □ |
| Critical Care | □ □ □ □ □ | □ □ □ □ □ |
| Dermatology | □ □ □ □ □ | □ □ □ □ □ |
| Endocrinology | □ □ □ □ □ | □ □ □ □ □ |
| Gastroenterology | □ □ □ □ □ | □ □ □ □ □ |
| Geriatric Medicine | □ □ □ □ □ | □ □ □ □ □ |
| Hematology | □ □ □ □ □ | □ □ □ □ □ |
| Immunology/Allergy | □ □ □ □ □ | □ □ □ □ □ |
| Infectious Diseases | □ □ □ □ □ | □ □ □ □ □ |
| Nephrology | □ □ □ □ □ | □ □ □ □ □ |
| Neurology | □ □ □ □ □ | □ □ □ □ □ |
| Medical Oncology | □ □ □ □ □ | □ □ □ □ □ |
| Palliative Medicine | □ □ □ □ □ | □ □ □ □ □ |
| Physical Medicine and Rehabilitation | □ □ □ □ □ | □ □ □ □ □ |
| Respirology | □ □ □ □ □ | □ □ □ □ □ |
| Rheumatology | □ □ □ □ □ | □ □ □ □ □ |

1. **Procedural Skills:** *We are not asking about core skills as they would be considered essential*.
2. How well **prepared** for practice did you feel in each of these procedures?
3. How **important** is skill in each of these procedures for GIM training now?
4. What is your **current** practice pattern?

## Check score in each column

|  | **1. Preparation** Score how well you felt prepared at the end of your GIM training | **2. Importance** Score for those currently in training | **3. How many times** a month do you perform this procedure? | **Where** learned if outside of residency. |
| --- | --- | --- | --- | --- |
|  | **1 = not at all**  **5 = very well** | **1 =not at all 5 = very** |  |  |
|  | **1 2 3 4 5** | **1 2 3 4 5** | **0 <5 5-9 10-14 15+** |  |
| ACLS/CPR | □ □ □ □ □ | □ □ □ □ □ | □ □ □ □ □ |  |
| Allergy testing | □ □ □ □ □ | □ □ □ □ □ | □ □ □ □ □ |  |
| Ambulatory ECG interpretation | □ □ □ □ □ | □ □ □ □ □ | □ □ □ □ □ |  |
| Articular drainage | □ □ □ □ □ | □ □ □ □ □ | □ □ □ □ □ |  |
| Bone marrow interpretation | □ □ □ □ □ | □ □ □ □ □ | □ □ □ □ □ |  |
| Bronchoscopy | □ □ □ □ □ | □ □ □ □ □ | □ □ □ □ □ |  |
| Cardioversion | □ □ □ □ □ | □ □ □ □ □ | □ □ □ □ □ |  |
| Chest tube insertion | □ □ □ □ □ | □ □ □ □ □ | □ □ □ □ □ |  |
| Colonoscopy | □ □ □ □ □ | □ □ □ □ □ | □ □ □ □ □ |  |
| Echocardiography | □ □ □ □ □ | □ □ □ □ □ | □ □ □ □ □ |  |
| Endotracheal intubation | □ □ □ □ □ | □ □ □ □ □ | □ □ □ □ □ |  |
| Esophagogastroscopy | □ □ □ □ □ | □ □ □ □ □ | □ □ □ □ □ |  |
| Exercise stress testing | □ □ □ □ □ | □ □ □ □ □ | □ □ □ □ □ |  |
| Hemodialysis | □ □ □ □ □ | □ □ □ □ □ | □ □ □ □ □ |  |
| Hemodynamic monitoring | □ □ □ □ □ | □ □ □ □ □ | □ □ □ □ □ |  |
| Indirect laryngoscopy | □ □ □ □ □ | □ □ □ □ □ | □ □ □ □ □ |  |
| Liver biopsy | □ □ □ □ □ | □ □ □ □ □ | □ □ □ □ □ |  |

b (continued). **Procedural Skills:** *We are not asking about core skills as they would be considered essential*.

1. How well **prepared** for practice did you feel in each of these procedures?
2. How **important** is skill in each of these procedures for GIM training now?
3. What is your **current** practice pattern?

**Check score in each column**

|  | **1. Preparation** Score for how well you felt prepared at the end of your GIM training | **2. Importance** Score for those currently in training | **3. How many times** a month do you perform this procedure? | **Where** learned if outside of residency. |
| --- | --- | --- | --- | --- |
|  | **1 = not at all**  **5 = very well** | **1 =not at all 5 = very** |  |  |
|  | **1 2 3 4 5** | **1 2 3 4 5** | **0 <5 5-9 10-14 15+** |  |
| Lumbar puncture | □ □ □ □ □ | □ □ □ □ □ | □ □ □ □ □ |  |
| Mechanical ventilation | □ □ □ □ □ | □ □ □ □ □ | □ □ □ □ □ |  |
| Paracentesis | □ □ □ □ □ | □ □ □ □ □ | □ □ □ □ □ |  |
| Peripheral smear interpretation | □ □ □ □ □ | □ □ □ □ □ | □ □ □ □ □ |  |
| Peritoneal dialysis | □ □ □ □ □ | □ □ □ □ □ | □ □ □ □ □ |  |
| Pleural biopsy | □ □ □ □ □ | □ □ □ □ □ | □ □ □ □ □ |  |
| Renal biopsy | □ □ □ □ □ | □ □ □ □ □ | □ □ □ □ □ |  |
| Sigmoidoscopy | □ □ □ □ □ | □ □ □ □ □ | □ □ □ □ □ |  |
| Sputum Gram stain | □ □ □ □ □ | □ □ □ □ □ | □ □ □ □ □ |  |
| Steroid injections (non-articular) | □ □ □ □ □ | □ □ □ □ □ | □ □ □ □ □ |  |
| Temporary pacemaker insertion | □ □ □ □ □ | □ □ □ □ □ | □ □ □ □ □ |  |
| Thyroid fine needle biopsy | □ □ □ □ □ | □ □ □ □ □ | □ □ □ □ □ |  |
| TPN initiation | □ □ □ □ □ | □ □ □ □ □ | □ □ □ □ □ |  |
| Tracheostomy tube change | □ □ □ □ □ | □ □ □ □ □ | □ □ □ □ □ |  |
| Transthoracic pacing | □ □ □ □ □ | □ □ □ □ □ | □ □ □ □ □ |  |
| Urine microscopic examination | □ □ □ □ □ | □ □ □ □ □ | □ □ □ □ □ |  |

1. **Skills Preparation**
   - 1. How well **prepared** for practice did you feel in each of these skills?
     2. How **important** is knowledge in each of these skills for GIM (post-core) training now?

## Check score in each column

|  | **1. Preparation** **Score**  for how well you felt prepared at the end of your GIM training | 2. Importance Score for those currently in training |
| --- | --- | --- |
|  | **1 =not at all 5=very well** | **1 = not at all 5 = very** |
|  | **1 2 3 4 5** | **1 2 3 4 5** |
| History taking | □ □ □ □ □ | □ □ □ □ □ |
| Physical examination | □ □ □ □ □ | □ □ □ □ □ |
| Diagnosis of undifferentiated illness | □ □ □ □ □ | □ □ □ □ □ |
| Management of chronic illness | □ □ □ □ □ | □ □ □ □ □ |
| Care of multi-system disease | □ □ □ □ □ | □ □ □ □ □ |
| Care of critically ill patients | □ □ □ □ □ | □ □ □ □ □ |
| Recognize treatable rare diseases. | □ □ □ □ □ | □ □ □ □ □ |
| Communication skills | □ □ □ □ □ | □ □ □ □ □ |
| Develop problem-solving | □ □ □ □ □ | □ □ □ □ □ |
| Develop life-long learning skills | □ □ □ □ □ | □ □ □ □ □ |
| Medical problems of pregnancy | □ □ □ □ □ | □ □ □ □ □ |
| Peri-operative care | □ □ □ □ □ | □ □ □ □ □ |
| Therapeutics | □ □ □ □ □ | □ □ □ □ □ |
| Critical appraisal | □ □ □ □ □ | □ □ □ □ □ |
| Consultation skills | □ □ □ □ □ | □ □ □ □ □ |
| Teaching skills | □ □ □ □ □ | □ □ □ □ □ |
| Research skills | □ □ □ □ □ | □ □ □ □ □ |
| Administration skills | □ □ □ □ □ | □ □ □ □ □ |
| Ethics | □ □ □ □ □ | □ □ □ □ □ |
| Set-up of an office | □ □ □ □ □ | □ □ □ □ □ |
| Counsel regarding smoking | □ □ □ □ □ | □ □ □ □ □ |
| Counsel regarding exercise | □ □ □ □ □ | □ □ □ □ □ |
| Counsel regarding HIV testing | □ □ □ □ □ | □ □ □ □ □ |
| Counsel about domestic violence | □ □ □ □ □ | □ □ □ □ □ |
| Counsel about substance abuse | □ □ □ □ □ | □ □ □ □ □ |
| Pain management | □ □ □ □ □ | □ □ □ □ □ |
| End-of-life issues | □ □ □ □ □ | □ □ □ □ □ |
| Compliance issues | □ □ □ □ □ | □ □ □ □ □ |
| Choose cost effective treatments | □ □ □ □ □ | □ □ □ □ □ |
| Participate in quality assurance | □ □ □ □ □ | □ □ □ □ □ |
| Collaborate with non-physician caregivers | □ □ □ □ □ | □ □ □ □ □ |

**d. Specific Disorders**

1. How well **prepared** for practice did you feel in each of following disorders?

2. How **important** is knowledge in each of these disorders for GIM (post-core) training **now**?

## Check score in each column

|  | **1. Preparation** **Score**  how well you felt prepared at the end of your GIM training | 2. Importance Score for those currently in training |
| --- | --- | --- |
|  | **1 = not at all 5 = very well** | **1 = not at all 5 = very** |
|  | **1 2 3 4 5** | **1 2 3 4 5** |
| Acute MI /acute coronary syndromes | □ □ □ □ □ | □ □ □ □ □ |
| Acute renal failure | □ □ □ □ □ | □ □ □ □ □ |
| Alcoholism | □ □ □ □ □ | □ □ □ □ □ |
| Anemia | □ □ □ □ □ | □ □ □ □ □ |
| Angina | □ □ □ □ □ | □ □ □ □ □ |
| Arrhythmias | □ □ □ □ □ | □ □ □ □ □ |
| Arthritis | □ □ □ □ □ | □ □ □ □ □ |
| Asthma | □ □ □ □ □ | □ □ □ □ □ |
| Cerebrovascular disease | □ □ □ □ □ | □ □ □ □ □ |
| Chronic obstructive pulmonary disease | □ □ □ □ □ | □ □ □ □ □ |
| Chronic renal failure | □ □ □ □ □ | □ □ □ □ □ |
| Collagen vascular disease | □ □ □ □ □ | □ □ □ □ □ |
| Congestive heart failure | □ □ □ □ □ | □ □ □ □ □ |
| Delirium | □ □ □ □ □ | □ □ □ □ □ |
| Dementia | □ □ □ □ □ | □ □ □ □ □ |
| Depression | □ □ □ □ □ | □ □ □ □ □ |
| Diabetes | □ □ □ □ □ | □ □ □ □ □ |
| Diabetic ketoacidosis | □ □ □ □ □ | □ □ □ □ □ |
| Electrolyte / acid base disorders | □ □ □ □ □ | □ □ □ □ □ |
| Headache | □ □ □ □ □ | □ □ □ □ □ |
| HIV/AIDS | □ □ □ □ □ | □ □ □ □ □ |
| Hyperlipidemia | □ □ □ □ □ | □ □ □ □ □ |
| Hypertension | □ □ □ □ □ | □ □ □ □ □ |
| Leukemia | □ □ □ □ □ | □ □ □ □ □ |
| Lymphoma | □ □ □ □ □ | □ □ □ □ □ |
| Malignancy | □ □ □ □ □ | □ □ □ □ □ |
| Overdose | □ □ □ □ □ | □ □ □ □ □ |
| Pyrexia of Unknown Origin | □ □ □ □ □ | □ □ □ □ □ |
| Substance abuse disorders | □ □ □ □ □ | □ □ □ □ □ |
| Thromboembolic disease | □ □ □ □ □ | □ □ □ □ □ |
| Thyroid dysfunction | □ □ □ □ □ | □ □ □ □ □ |
| Vasculitis | □ □ □ □ □ | □ □ □ □ □ |

1. **Other elements of the GIM (post-core) training program**

1. How well did your training program **meet** your needs in terms of these other elements.

2. How **important** you feel each of the elements is in the design of a training program for current trainees?

## Check score in each column

|  | 1. How well did the training program **meet** your needs in this domain? | 2. How **important** is this domain to current trainees? |
| --- | --- | --- |
|  | **1 = not at all 5 = very well** | **1 = not at all 5 = very** |
|  | **1 2 3 4 5** | **1 2 3 4 5** |
| Acute care | □ □ □ □ □ | □ □ □ □ □ |
| Perioperative care | □ □ □ □ □ | □ □ □ □ □ |
| Peripartum care | □ □ □ □ □ | □ □ □ □ □ |
| Critical care – ICU | □ □ □ □ □ | □ □ □ □ □ |
| Critical care – CCU | □ □ □ □ □ | □ □ □ □ □ |
| Chronic care | □ □ □ □ □ | □ □ □ □ □ |
| Research skills | □ □ □ □ □ | □ □ □ □ □ |
| Administration skills | □ □ □ □ □ | □ □ □ □ □ |
| Quality assurance/improvement | □ □ □ □ □ | □ □ □ □ □ |
| Computer and internet use | □ □ □ □ □ | □ □ □ □ □ |
| Exposure to procedural skills | □ □ □ □ □ | □ □ □ □ □ |
| Journal clubs | □ □ □ □ □ | □ □ □ □ □ |
| Seminars | □ □ □ □ □ | □ □ □ □ □ |
| Opportunity for self-directed learning | □ □ □ □ □ | □ □ □ □ □ |
| Directed readings | □ □ □ □ □ | □ □ □ □ □ |
| Flexibility of programme | □ □ □ □ □ | □ □ □ □ □ |
| Administrative structure | □ □ □ □ □ | □ □ □ □ □ |
| Clear goals and objectives | □ □ □ □ □ | □ □ □ □ □ |
| Increasing professional responsibility | □ □ □ □ □ | □ □ □ □ □ |
| Responsibility separate from that of a third year resident | □ □ □ □ □ | □ □ □ □ □ |
| Timely on-going evaluation and feedback | □ □ □ □ □ | □ □ □ □ □ |
| Number and variety of Patients | □ □ □ □ □ | □ □ □ □ □ |
| Ambulatory care rotations | □ □ □ □ □ | □ □ □ □ □ |
| In-patient rotations | □ □ □ □ □ | □ □ □ □ □ |
| GIM consultation services | □ □ □ □ □ | □ □ □ □ □ |
| Community GIM rotations | □ □ □ □ □ | □ □ □ □ □ |

**2. Length of GIM (Post-core) Training:**

a. How many years do you believe GIM post-core training should be?

- One Year
- Two Years
- Other: please state number of years ___________

b. If you were in a **2 year** post-core GIM training program do you believe that this was:

- Too Long
- Just Right
- Too Short
- Not applicable

c. If you were in a **1 year** post-core GIM training program do you believe that this was:

- Too Long
- Just Right
- Too Short
- Not applicable

d. Year of completion of General Internal Medicine (Post Core) Training: ____

e. University at which training was taken: _________________

f. Number of years of GIM post-core residency training: 1 □ 2 □ >2 □

g. Number of years of other subspecialty post core residency training:

0 □ 1 □ 2 □ 3 □ >3 □

h. Name of subspecialty: _________________

#### 3. Demographics and Present Practice Please give us a picture of your practice.

**a. Are you still in a training program?** yes **□** no □

If yes, proceed to question 5.

To describe your present practice, check all relevant words or phrases below or briefly describe in narrative format your clinical and non-clinical activities & interests.

**b. Practice type**:

□ community-based without a university appointment

□ community-based with a university appointment

□ university-based full-time

□ other(specify): _______________________

**c. in-patient care:**

□ consultation □ primary care □ both □ neither

**d. out-patient care**:

□ consultation □ primary care □ both □ neither

**e. location**: □ hospital-based □ office-based □ both

□ other (specify): ___________________________

**f. catchment area:**

□ rural

□ small (<100,000) urban

□ large (>100,000) urban

□ >100 km from tertiary care centre

□ other(describe): ________________________________

**g. clinical practice:**

□ >75% General Internal Medicine

**h. special clinical 'interest'** in:

□ ICU □ CCU □ cardiology □ gastroenterology □ diabetes

□ endocrinology □ respirology □ medical problems in pregnancy

□ pre-operative consultation □ other (specify): ____________________________

**i. specific procedure training** in (specify): ____________________ duration (months) : __

**j. other training or interest:**

□ clinical epidemiology □ medical education □ public health □health services □ ethics □ health administration □ other (specify): ________________________

**k. 'non clinical' activities** (provide % of total work time spent in each):

teaching ___ %

research ____%

administration _____ %

other ______ % (describe): _____________

##### 4. Please provide any other information to describe your practice

**5. Please add any comments or suggestions for training in General Internal Medicine**

***MANY THANKS FOR COMPLETING THIS SURVEY!!!***
